# TGF-β1 enhances FOXO3 expression in human synovial fibroblasts by inhibiting miR-92a through AMPK and p38 pathways

**DOI:** 10.18632/aging.102038

**Published:** 2019-06-21

**Authors:** Shu-Jui Kuo, Shan-Chi Liu, Yuan-Li Huang, Chun-Hao Tsai, Yi-Chin Fong, Horng-Chaung Hsu, Chih-Hsin Tang

**Affiliations:** 1 School of Medicine, China Medical University, Taichung, Taiwan; 2 Department of Orthopedic Surgery, China Medical University Hospital, Taichung, Taiwan; 3 Department of Medical Education and Research, China Medical University Beigang Hospital, Yunlin, Taiwan; 4 Department of Biotechnology, College of Health Science, Asia University, Taichung, Taiwan; 5 Department of Sports Medicine, College of Health Care, China Medical University, Taichung, Taiwan; 6 Chinese Medicine Research Center, China Medical University, Taichung, Taiwan

**Keywords:** osteoarthritis, TGF-β1, FOXO3, miRNA-92a, synovial fibroblasts

## Abstract

Osteoarthritis (OA) is an age-related disease marked by synovial inflammation and cartilage destruction arising from synovitis, joint swelling and pain. OA therapy that targets the synovium is a promising strategy for mitigating the symptoms and disease progression. Altered activity of the transforming growth factor-β1 isoform (TGF-β1) during aging underlies OA progression. Notably, aberrant forkhead box class O 3 (FOXO3) activity is implicated in the pathogenesis of various age-related diseases, including OA. This study explored the interaction and cross-talk of TGF-β1 and FOXO3 in human osteoarthritis synovial fibroblasts (OASFs). TGF-β1 stimulated FOXO3 synthesis in OASFs, which was mitigated by blocking adenosine monophosphate-activated protein kinase (AMPK) and p38 activity. TGF-β1 also inhibited the expression of miR-92a, which suppresses FOXO3 transcription. The suppression of miR-92a was effectively reversed with the blockade of the AMPK and p38 pathways. Our study showed that TGF-β1 promotes anti-inflammatory FOXO3 expression by stimulating the phosphorylation of AMPK and p38 and suppressing the downstream expression of miR-92a. These results may help to clarify OA pathogenesis and lead to better targeted treatment.

## Introduction

Osteoarthritis (OA) is marked by synovial inflammation, cartilage destruction, joint swelling and pain. Age-associated inflammation is a key contributor to the pathogenesis of OA [1,2], as a result of continuous mechanical wear and tear and/or age-related modifications of the cartilage matrix. Cellular apoptosis and senescence potentially also contribute to the progression of OA [3,4].

The synovium plays an integral role in OA. The synthesis of pro-inflammatory and hydrolytic mediators by the inflamed synovium can induce cartilage erosion, which amplifies synovial inflammation, creating a vicious cycle. OA synovial cells maintain arthritic pathologies by synthesizing the inflammatory mediators and matrix degradation enzymes [5–7]. Thus, research has begun to focus on synovium-targeted therapy in the attempt to halt the progression and lessen the impact of OA symptoms [8,9].

The homodimeric protein, transforming growth factor beta 1 (TGF-β1), binds to TGF-β type I (TGFBR1) and type II (TGFBR2) serine/threonine kinase receptors on the cell membranes of the target cells via Smad-dependent and Smad-independent pathways [6]. TGF-β1 signaling is altered during aging and is a driving force of OA progression [10,11]. TGF-β1 concentrations differ substantially between healthy and OA joints, and activate different signaling pathways [10]. To date, the differential signaling pathways between healthy and OA joints have not been fully understood. The forkhead box class O (FOXO) transcription factors are among the major mediators that regulate cellular aging and stress resistance. The mammalian FOXO family (i.e., FOXO1, FOXO3 and FOXO4) [12] regulates the expression of genes governing oxidative defense and DNA repair enzymes, and modulates the activity of the ubiquitin–proteasome system and the autophagic/lysosomal pathway [13–16]. Aberrant FOXO activity is found in the pathogenesis of various age-related diseases, including OA [17–19].

Non-coding, single-stranded micro-ribonucleic acids (miRNAs) modulate the expression of target genes at the post-transcriptional level [20,21]. By base pairing with the seed sequence of target mRNA molecules, the 3'-untranslated region (3'-UTR), miRNAs inhibit the expression of target genes. Various miRNAs are involved in OA pathogenesis [22,23]. Despite TGF-β1 and FOXO3 activity being similarly involved in the pathogenesis of OA as well as the aging process, no investigations to date have evaluated any possible correlations between TGF-β1 and FOXO3. In view of the importance of synovial cells in OA pathogenesis, we explored the interaction and cross-talk between TGF-β1 and FOXO3 in human osteoarthritis synovial fibroblasts (OASFs). We hypothesized that TGF-β1 upregulates FOXO3 expression by modulating intermediate miRNA expression in OASFs.

## RESULTS

### TGF-β1 stimulates the expression of FOXO3 in human OASFs

**Figure 1:**
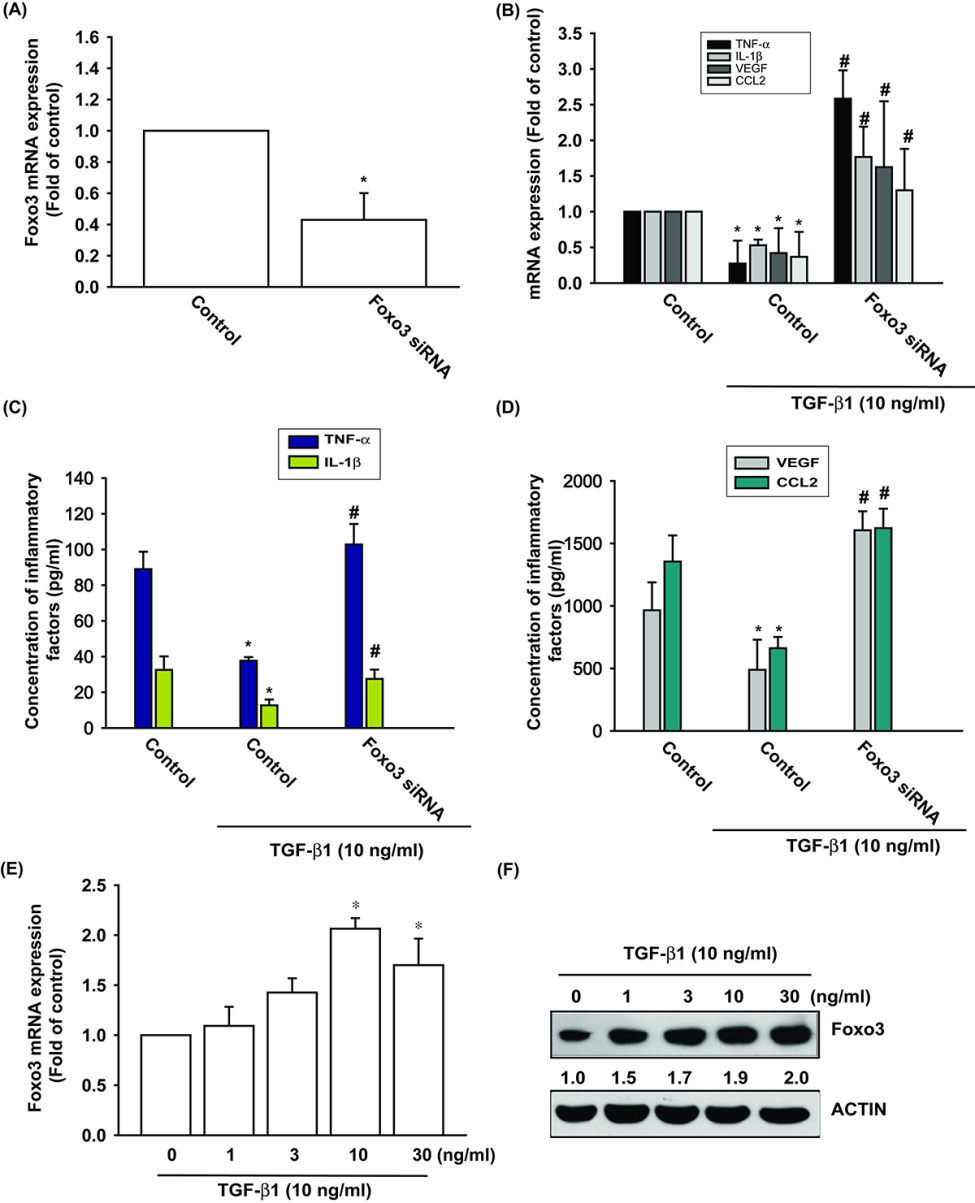
**TGF-β1 promotes anti-inflammatory effects via FOXO3-dependent expression in OASFs.** (**A**) OASFs were transfected with FOXO3 siRNA and the FOXO3 mRNA expression was examined by qPCR. (**B-D**) OASFs were transfected with FOXO3 siRNA, then incubated with TGF-β1 (10 ng/ml). The mRNA and protein levels of TNF-α, IL-1β, VEGF and CCL2 was examined by qPCR and ELISA. OASFs were incubated with 0, 1, 3, 10, and 30 ng/ml of TGF-β1 for 24 h; FOXO3 mRNA and protein expression were examined by qPCR (**E**) and Western blot (**F**). Results are expressed as the mean ± SEM. *p < 0.05 as compared with the control group; #*p* < 0.05 as compared with the TGF-β1-treated group.

We have already established that TGF-β1 promotes levels of anti-inflammatory enzyme HO-1 expression in OASFs [6]. We therefore examined the anti-inflammatory effects of TGF-β1. Stimulation of OASFs with TGF-β1 diminished the mRNA and protein expression of inflammatory mediators, including TNF-α, IL-1β, VEGF and CCL2 (Fig. 1B-D). Both TGF-β1 and FOXO3 have been shown to be involved in the pathogenesis of OA, as well as the aging process [10,19]. However, the interplay between TGF-β1 and FOXO3 in the pathogenesis of OA and their impact on OASFs is unclear. Transfection of cells with FOXO3 siRNA suppressed FOXO3 expression (Fig. 1A) and reversed TGF-β1-inhibited the mRNA and protein expression of TNF-α, IL-1β, VEGF and CCL2 (Fig. 1B-D). We also found that TGF-β1 (0–30 ng/ml) stimulated the synthesis of FOXO3 mRNA and protein in a concentration-dependent manner (Fig. 1E and 1F). According to these data, TGF-β1 promotes anti-inflammatory effects through FOXO3 expression.

### TGF-β1 stimulates FOXO3 expression via the phosphorylation of AMP activated protein kinase (AMPK) and p38

**Figure 2:**
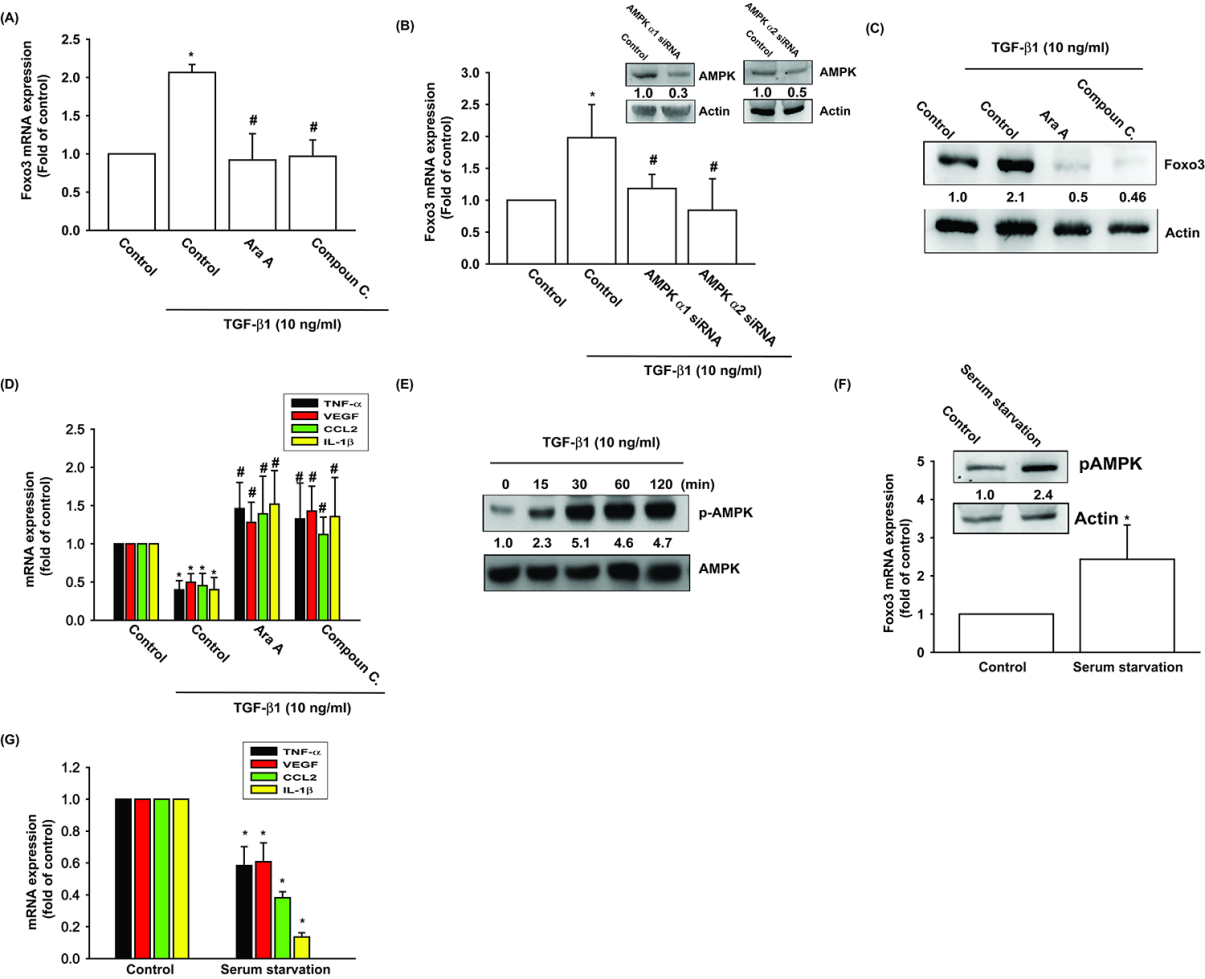
**AMPK activation is involved in TGF-β1-induced FOXO3 synthesis.** (**A-D**) OASFs were pretreated with AMPK inhibitors (Ara A and compound C) or transfected with AMPKα1 and α2 siRNAs, then incubated with TGF-β1 (10 ng/ml). The mRNA and protein levels were examined by qPCR and Western blot. (**E**) OASFs were incubated with TGF-β1 for the indicated time intervals, and the extent of AMPK phosphorylation was examined by Western blot. (**F**) Cells were serum starvation for 24 h, the AMPK phosphorylation and indicated mRNA expression were examined by Western blot and qPCR. Results are expressed as the mean ± SEM. *p < 0.05 as compared with the control group; #*p* < 0.05 as compared with the TGF-β1-treated group.

The AMP activated protein kinase (AMPK) is regulated by various stimuli, including TGF-β1 [24]. To validate the role of AMPK in TGF-β1-enhanced FOXO3 production, we pretreated OASFs with AMPK inhibitors (Ara A and compound C) or transfected them with AMPK α1/α2 siRNAs. The qPCR and Western blot assay confirmed significant mitigation of TGF-β1-enhanced FOXO3 synthesis in OASFs after the administration of AMPK inhibitors and AMPK α1/α2 siRNAs (Fig. 2A-C). AMPK inhibitors also reversed TGF-β1-inhibited the expression inflammatory mediators (Fig. 2D). TGF-β1-induced stimulation of OASFs led to a time-dependent increase in the phosphorylation of AMPK, as shown by Western blot (Fig. 2E). Increasing the AMP:ATP ratio leads to activate AMPK signaling. We also found serum starvation increased AMPK phosphorylation and FOXO3 synthesis as well as suppressed the expression of inflammatory mediators (Fig. 2F, G).

**Figure 3:**
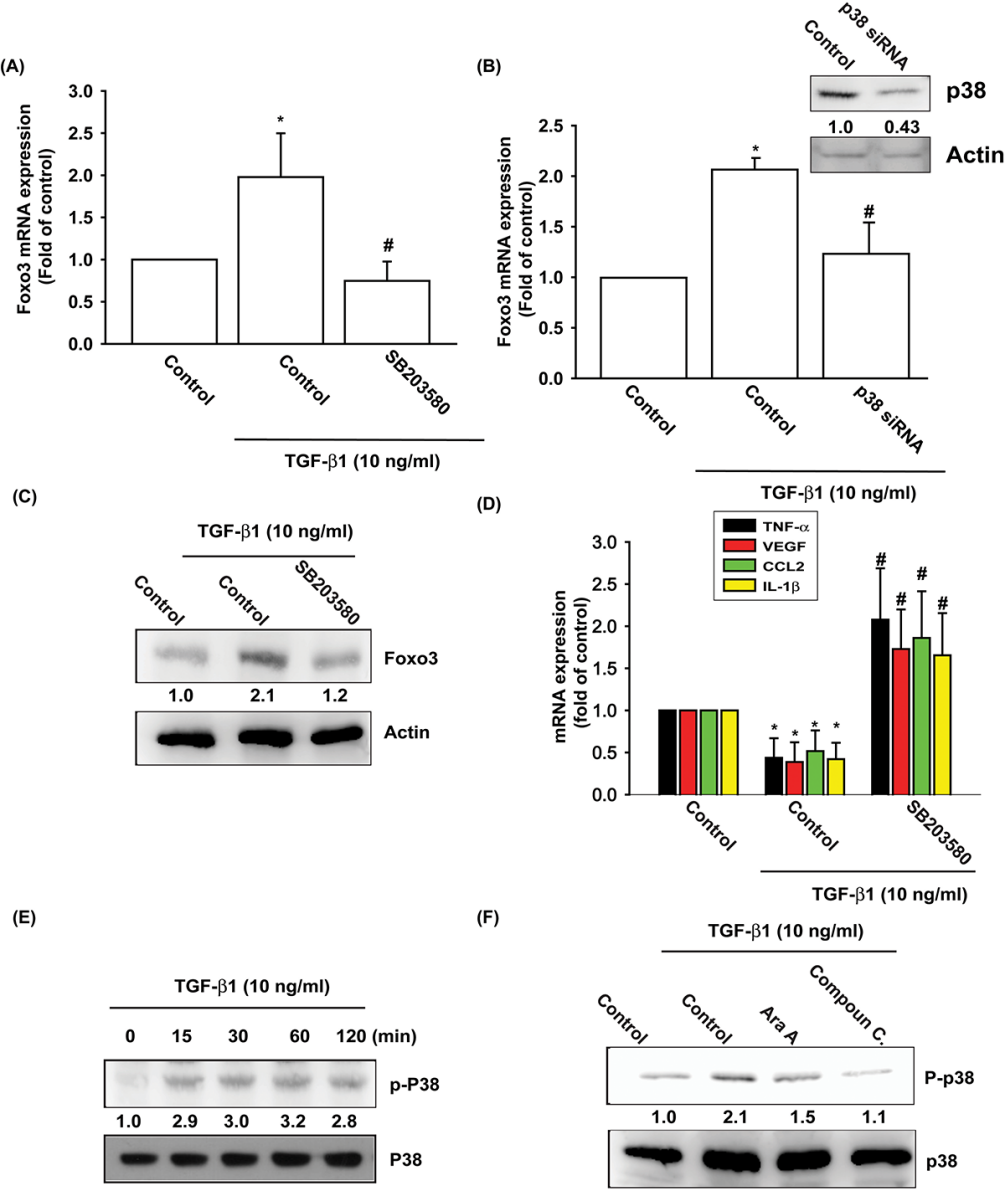
**The p38 pathway is involved in TGF-β1-induced FOXO3 production.** (**A-D**) OASFs were pretreated with a p38 inhibitor (SB203580) or transfected with p38 siRNA for 24 h, then incubated with TGF-β1 (10 ng/ml) for 24 h. The mRNA and protein levels were examined by qPCR and Western blot. (**E**) OASFs were incubated with TGF-β1 for the indicated time intervals; the extent of p38 phosphorylation was examined by Western blot. (**F**) OASFs were pretreated with AMPK inhibitors for 24 h, then incubated with TGF-β1 (10 ng/ml). The p38 phosphorylation was examined by Western blot. Results are expressed as the mean ± SEM. *p < 0.05 as compared with the control group; #*p* < 0.05 as compared with the TGF-β1-treated group.

The p38, a mitogen-activated protein kinase involved in cell differentiation, aging and autophagy, regulates chondrocyte apoptosis and is involved in OA pathogenesis [25,26]. AMPK stimulates downstream p38 activity [27,28]. We pretreated OASFs with a p38 inhibitor (SB203580) and p38 siRNA prior to TGF-β1 administration. As shown in Fig. 3A-D, pretreatment with SB203580 or transfection with p38 siRNA significantly mitigated TGF-β1-enhanced FOXO3 synthesis and TGF-β1-inhibited the expression of inflammatory mediators. Under Western blot assay, TGF-β1 time-dependently stimulated the phosphorylation of p38 (Fig. 3E), and the TGF- β 1-induced p38 phosphorylation mitigated by AMPK inhibitors (Fig. 3F). These data demonstrate that TGF-β1 enhances FOXO3 expression through AMPK and p38 signaling pathways.

### TGF-β1 enhances FOXO3 expression by inhibiting miR-92a synthesis

**Figure 4:**
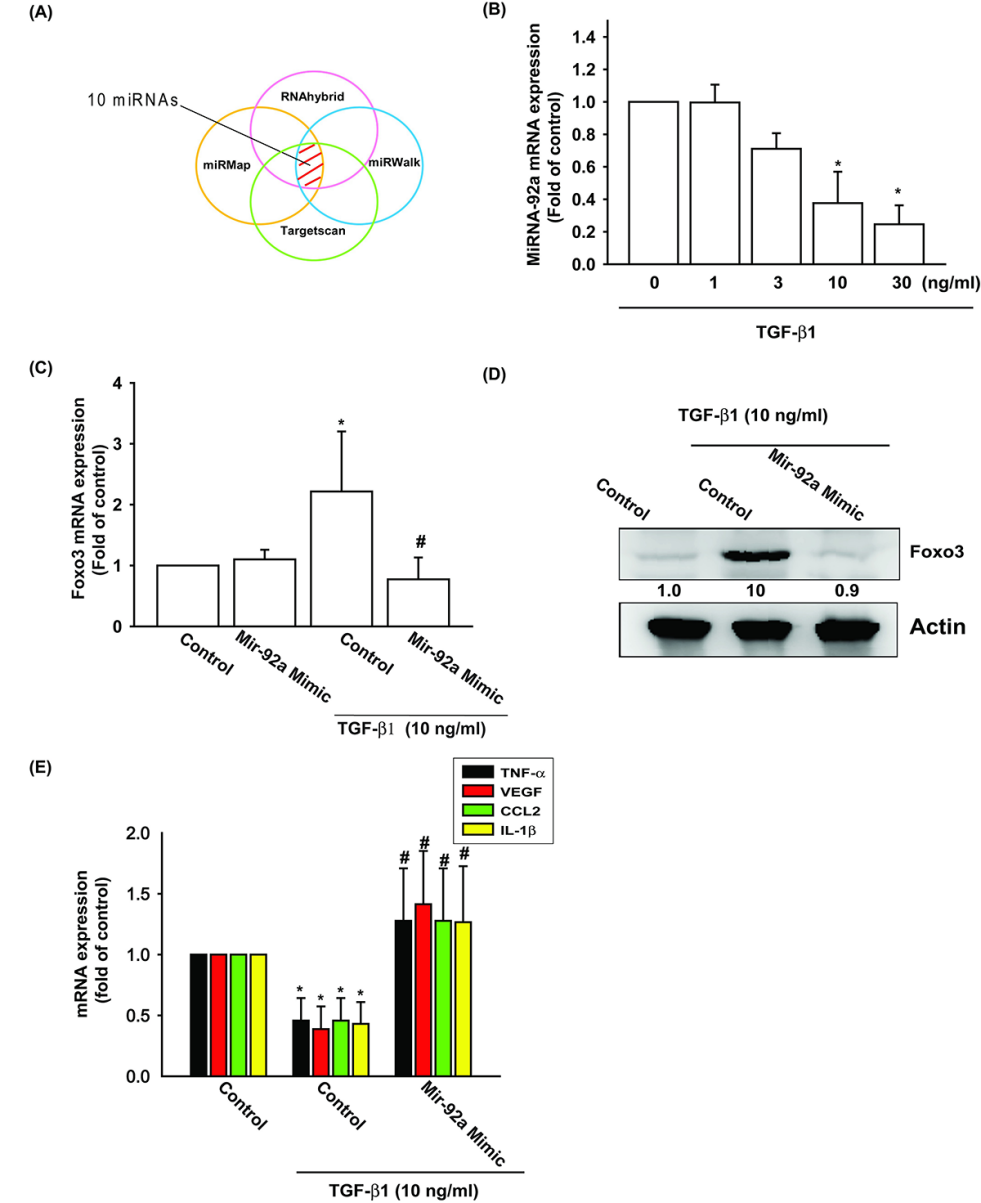
**TGF-β1 suppression of miR-92a enhances FOXO3 production.** (**A**) Open-source software (TargetScan, miRDB, and miRWalk) was used to identify which miRNAs could possibly interfere with FOXO3 transcription. (**B**) OASFs were incubated with TGF-β1 at concentrations of 0, 1, 3, 10, and 30 ng/ml. miR-92a expression levels were examined by qPCR assay. (**C-E**) OASFs were transfected with miR-92a mimic and then stimulated with TGF-β1. The mRNA and protein levels were examined by qPCR and Western blot. Results are expressed as the mean ± SEM. *p < 0.05 as compared with the control group; #*p* < 0.05 as compared with the TGF-β1-treated group.

Several miRNAs exhibit differential expression patterns between osteoarthritic and normal cartilage and are involved in the inflammatory and catabolic processes of OA [29]. However, the exact roles of miRNAs in OA pathogenesis are little understood. We used open-source software (TargetScan, miRDB, and miRWalk) to identify miRNAs that could possibly interfere with FOXO3 transcription (Fig. 4A). Among the 10 candidate miRNAs that could possibly bind to the 3’UTR region of FOXO3 mRNA, the expression level of miR-92 was most significantly suppressed after TGF-β1 administration (Supplementary Fig. S1). To confirm these findings, we compared levels of miR-92a expression in OASFs with different TGF-β1 dosages. We found that TGF-β1 (1–30 ng/ml) inhibited miR-92a expression in a concentration-dependent manner (Fig. 4B). To further determine whether TGF-β1 stimulates FOXO3 expression by inhibiting miR-92a synthesis, we transfected OASFs with the miR-92a mimic, which reduced TGF-β1-enhanced FOXO3 mRNA and protein synthesis (Fig. 4C and 4D). In addition, miR-92a mimic also antagonized TGF-β1-suppressed the expression of inflammatory mediators (Fig. 4E). These findings suggest that TGF-β1 promotes FOXO3 expression by suppressing miR-92a expression in OASFs.

**Figure 5:**
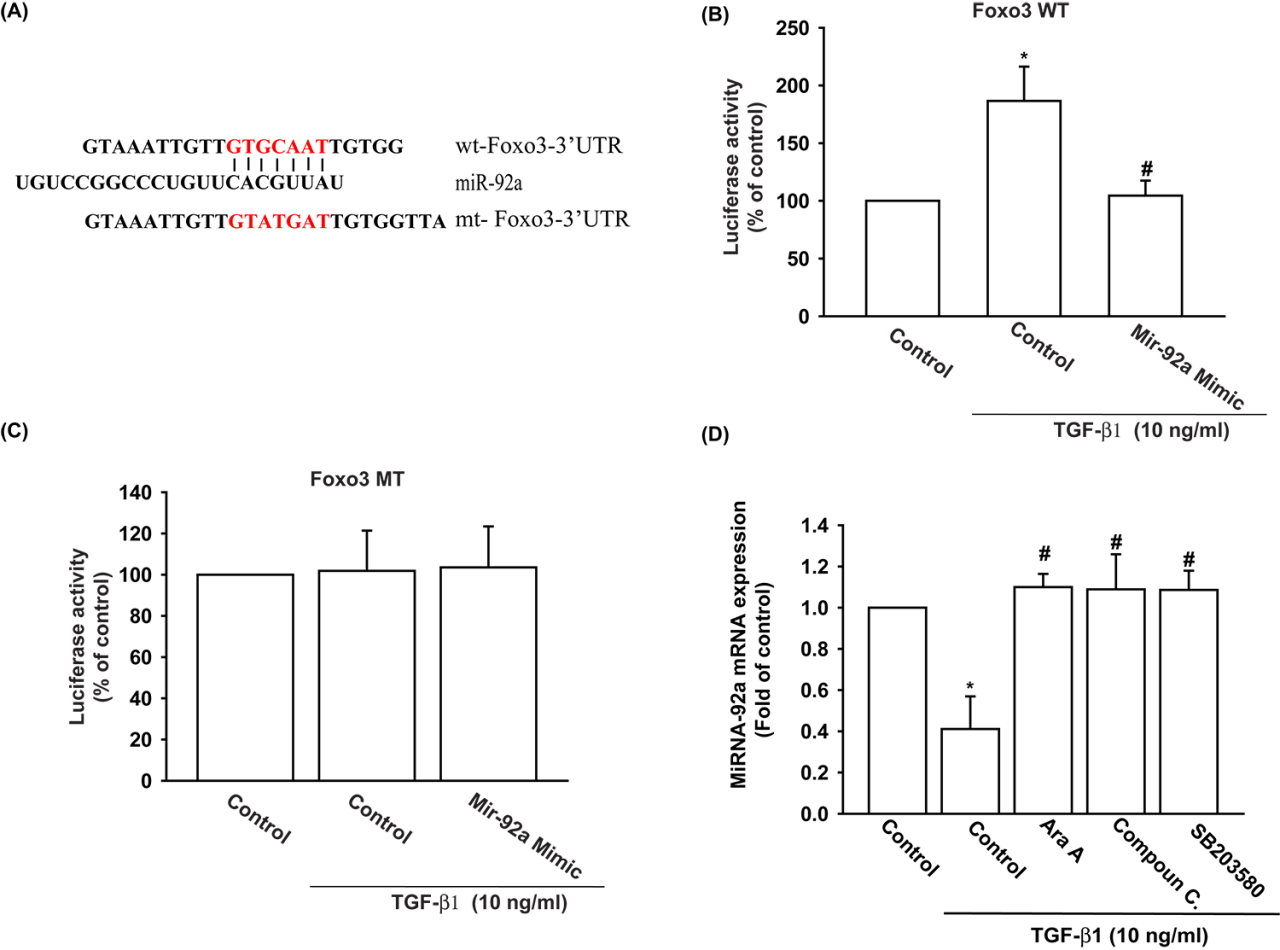
**The binding of miR-92a to FOXO3 3' UTR mitigates TGF-β1-induced increases in FOXO3 expression.** (**A**) Diagram of the miR-92a binding site in the wild-type and mutant FOXO3 3’UTRs. (**B, C**) OASFs were transfected with the wt-FOXO3-3’UTR (**B**) or mt-FOXO3-3’UTR (**C**) plasmid with or without miR-92a mimic, then stimulated with TGF-β1. FOXO3 promoter activity was expressed as the relative luciferase activity. (**D**) OASFs were pretreated with Ara A, compound C and SB203580 for 30 min, then incubated with TGF-β1 (10 ng/ml) for 24 h. The expression of miR-92a was examined by qPCR. Results are expressed as the mean ± SEM. *p < 0.05 as compared with the control group; #*p* < 0.05 as compared with the TGF-β1-treated group.

We also used the luciferase reporter vector, including the wild-type 3’UTR of FOXO3 mRNA (wt-FOXO3-3’UTR) and the vector harboring mismatches in the predicted miR-92a binding site (mt-FOXO3-3’UTR), to determine whether miR-92a regulates transcription of the *FOXO3* gene (Fig. 5A). We showed that miR-92a mimic reduced TGF-β1-enhanced luciferase activity in the wt-FOXO3-3’UTR plasmid, but not in the mt-FOXO3-3’UTR plasmid (Fig. 5B and 5C). In addition, the AMPK inhibitors (Ara A and compound C) and the p38 inhibitor (SB203580) reversed TGF-β1-inhibited miR-92a expression (Fig. 5D). It appears that miR-92a directly suppresses FOXO3 transcription by binding to the 3'UTR region of the human FOXO3 mRNA, and that miR-92a expression is negatively regulated by AMPK and p38 phosphorylation induced by upstream TGF-β1 signaling.

### TGF-β1 expression is positively correlated with FOXO3 expression in OA patients

**Figure 6:**
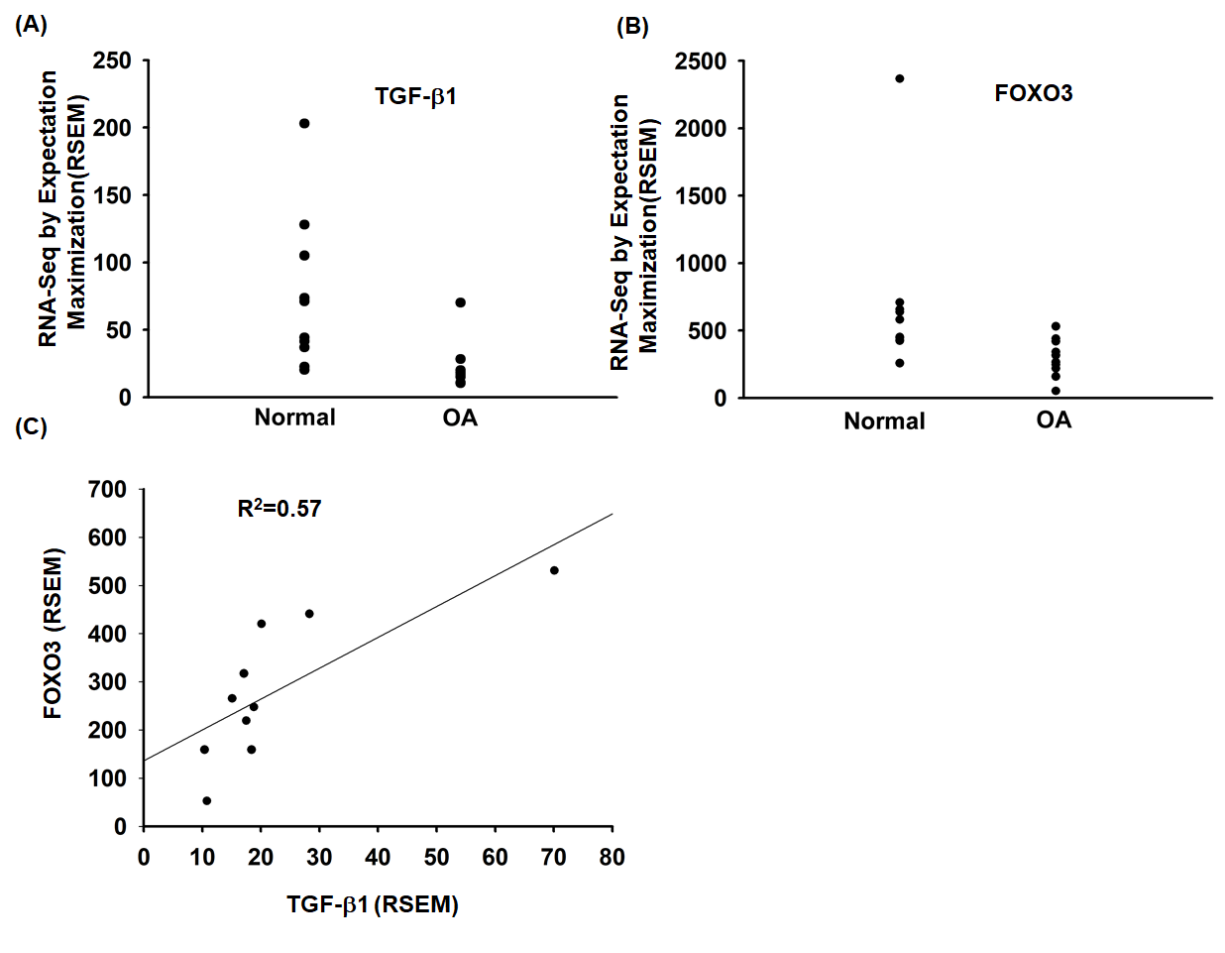
**The TGF-β1 and FOXO3 expression in OA patients.** (**A**, **B**) Expression levels of TGF-β1 and FOXO3 in paired normal and OA tissues retrieved from the GEO dataset records (GDS5403). (**C**) Correlation between TGF-β1 and FOXO3 expression levels in OA specimens retrieved from the GEO dataset.

To investigate the role of TGF-β1 and FOXO3 in OA patients, we analyzed the GEO database, which revealed lower levels of TGF-β1 and FOXO3 expression in OA tissue compared with normal tissue (Fig. 6A and 6B). Under immunohistochemistry staining, the expression of FOXO3 is substantially lower in OA tissue (Supplementary Fig. S2). In addition, TGF-β1 expression was positively correlated with FOXO3 expression in OA patients (Fig. 6C). Otherwise, the expression level of miR-92a was higher in OA tissue (Supplementary Fig. S3).

## DISCUSSION

TGF-β1 is a pleiotropic cytokine that is pivotal in the pathogenesis of OA. TGF-β1 concentrations differ substantially between healthy and OA joints, leading to the activation of different signaling pathways [10]. TGF-β1 can mediate the activities of chondrocytes, synovial cells and subchondral osteoblasts, and thus exert a global impact on the knee [30]. In spite of the global influence of TGF-β1 in the pathogenesis of OA, the impact of TGF-β1 on OASFs has not yet been fully delineated. Our team has previously discovered that TGF-β1 stimulates the expression of HO-1 in OASFs via the stimulation of PKCα phosphorylation and suppression of the downstream expression of miR-519b [6]. We found that TGF-β1 can stimulate anti-inflammatory gene FOXO3 expression in OASFs by stimulating AMPK and p38 phosphorylation and suppressing the downstream expression of miR-92a. These results support the existing literature on OA pathogenesis.

The miRNAs are small, non-coding RNA fragments that suppress the translation of or induce the degradation of target mRNAs [31]. Many miRNAs are involved in OA pathogenesis [22,32]. We used open-source software (TargetScan, miRDB, and miRWalk) to evaluate which candidate miRNAs can possibly interfere with the transcription of FOXO3. Among the selected miRNAs, miR-92a was suppressed to the greatest extent by TGF-β1 in a qPCR assay. We have shown that transfection of OASFs with miR-92a mimic mitigates TGF-β1-stimulated FOXO3 expression. These findings underscore the importance of miR-92a in the process of TGF-β1-stimulated FOXO3 expression.

The role of the AMPK and p38 in OA pathogenesis has been discussed previously. AMPK is a eukaryotic heterotrimeric serine/threonine protein kinase [33]. AMPK is activated by the increased intracellular AMP:ATP ratio, which enables cells to accommodate to changes in energy demands [33]. Decreased AMPK activity in articular chondrocytes as a result of aging, joint injury and chronic inflammation, leads to mitochondrial dysfunction, increased oxidative stress and matrix loss due to inflammation, as well as poor cellular quality control due to perturbed autophagy. These events typically lead to cell death and cartilage destruction, which enables the development and progression of OA [33,34]. Our previous research revealed that the phosphorylation of AMPK and p38 is involved in the adiponectin-enhanced interleukin-6 expression in OASFs [35]. In the present study, we show that TGF-β1 stimulates FOXO3 expression via AMPK and p38 phosphorylation. Our data emphasize the importance of AMPK and p38 in OA pathogenesis. It has been reported that transcriptional and posttranscriptional regulation play key role in miRNA activation and inhibition [36]. In this study, OASFs treatment with AMPK and p38 inhibitor antagonized TGF-β1-inhibited miR-92a expression indicating TGF-β1 reduced miR-92a expression through AMPK/p38 pathway. Whether AMPK/p38 regulate miR-92a expression through transcriptional or posttranscriptional regulation is needs further examination.

## Conclusion

**Figure 7:**
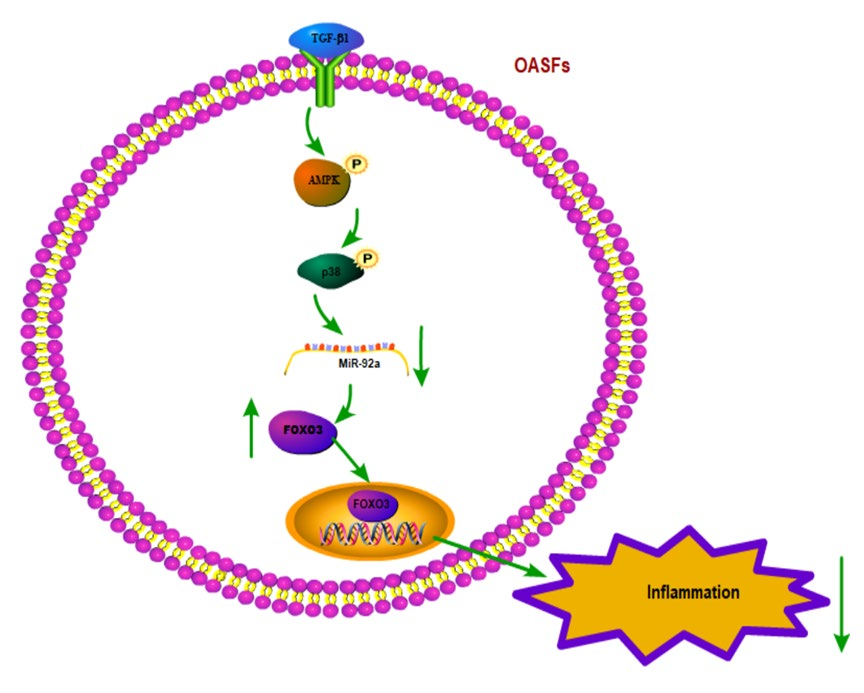
**Schematic diagram summarizes the mechanism whereby TGF-β1 promotes FOXO3 expression in OASFs.** TGF-β1 promotes anti-inflammatory FOXO3 expression in OASFs by downregulating miR-92a through the AMPK and p38 signaling pathways.

In summary, our study shows that the TGF-β1 in OASFs triggers the phosphorylation of AMPK and p38 and contributes to a decline in miR-92a synthesis. The decreased miR-92a expression enhances synthesis of the anti-inflammatory gene *FOXO3* (Fig. 7). These results improve our understanding about the role of OASFs in the pathogenesis of aging and OA and may result in more effective therapy for OA patients.

## MATERIALS AND METHODS

Antibodies against FOXO3, AMPK, and p38 were all purchased from Santa Cruz (Santa Cruz, CA, USA). Antibodies against p-AMPK and p-p38 were purchased from Cell Signaling (Cell Signaling, UK). ON-TARGETplus siRNAs for FOXO3, AMPKα1, AMPKα2, p38 and the control were bought from Dharmacon (Lafayette, CO, USA). Ara A, Compound C and SB203580 were supplied by Calbiochem (San Diego, CA, USA). Cell culture supplements were purchased from Invitrogen (Carlsbad, CA, USA). Dual-Luciferase^®^ Reporter Assay System was bought from Promega (Madison, WI, USA). The qPCR primers and probes, as well as the Taqman^®^ one-step PCR Master Mix, were supplied by Applied Biosystems (Foster City, CA). All other chemicals not described above were supplied by Sigma-Aldrich (St Louis, MO, USA).

### Cell culture

Synovial tissue from the suprapatellar pouch of the knee were obtained from 20 patients diagnosed as having stage IV OA. The cells were cultured in DMEM medium supplemented with 10% fetal bovine serum (FBS), 50 units/mL penicillin and 50 μg/mL streptomycin, as previously described [37,38]. The protocol was approved by the Institutional Review Board (IRB) of China Medical University Hospital and all methods were carried out in accordance with the IRB’s guidelines and regulations. Informed written consent was obtained from all patients.

### Real-time quantitative PCR of mRNA and miRNA

Total RNA was extracted from human synovial fibroblasts by TRIzol; reverse transcription used 1 μg of total RNA transcribed into cDNA by oligo (dT) primers [39,40]. Real-time quantitative PCR (RT-qPCR) used the Taqman® One-Step RT-PCR Master Mix. qPCR assays were then detected by StepOnePlus system.

### Western blot analysis

Cell lysate was separated by SDS-PAGE electrophoresis then transferred to polyvinylidene difluoride (PVDF) membranes, according to the method described in our previous studies [39,41]. After blocking the blots with 4% bovine serum albumin, the blots were treated with primary antibody and then secondary antibody. Enhanced chemiluminescent imaging of the blots was visualized with the UVP Biospectrum system (UVP, Upland, CA, USA).

### Analysis of the gene expression omnibus (GEO) dataset

Data for normal healthy controls and OA patients (n=10 in each group) were retrieved from the GEO (accession code; GDS5403) dataset, which contained mRNA sequencing.

### Plasmid construction and luciferase assays

Wild-type and mutant FOXO3 3′-UTRs were generated on the miR-92a target recognition sites (seed sequences). The wild-type 3′-UTRs of FOXO3 were cloned into the pmirGLO-luciferase reporter vector using NheI and BglII restriction sites. The primer sequences used were defined as FOXO3 forward primer: CGGCTAGCTGCGCCTTGGCTTTATAACT; the reverse primer: GGCTCGAGCCCTCCTTCACTGCTACTGG. All constructs were sequenced to verify that they contained the 3′-UTR inserts. The mutant 3′UTR region of FOXO3 mRNA (mt-FOXO3- 3′-UTR) was purchased from Invitrogen. Luciferase activity was assayed using the method described in our previous publications [42,43].

### Statistics

All values are given as the mean ± S.E.M. Between-group differences were assessed for significance using the Student’s *t-*test. The statistical difference was considered to be significant if the *p* value was <0.05.

## SUPPLEMENTARY MATERIAL

Supplementary Figures

## References

[1] Loeser RF. The Role of Aging in the Development of Osteoarthritis. Trans Am Clin Climatol Assoc. 2017; 128:44–54. 28790486 PMC5525396

[2] Greene MA, Loeser RF. Aging-related inflammation in osteoarthritis. Osteoarthritis Cartilage. 2015; 23:1966–71. 10.1016/j.joca.2015.01.008 26521742 PMC4630808

[3] Aigner T, Haag J, Martin J, Buckwalter J. Osteoarthritis: aging of matrix and cells--going for a remedy. Curr Drug Targets. 2007; 8:325–31. 10.2174/138945007779940070 17305510

[4] MacDonald IJ, Liu SC, Su CM, Wang YH, Tsai CH, Tang CH. Implications of Angiogenesis Involvement in Arthritis. Int J Mol Sci. 2018; 19:E2012. 10.3390/ijms19072012 29996499 PMC6073145

[5] Benito MJ, Veale DJ, FitzGerald O, van den Berg WB, Bresnihan B. Synovial tissue inflammation in early and late osteoarthritis. Ann Rheum Dis. 2005; 64:1263–67. 10.1136/ard.2004.025270 15731292 PMC1755629

[6] Kuo SJ, Yang WH, Liu SC, Tsai CH, Hsu HC, Tang CH. Transforming growth factor β1 enhances heme oxygenase 1 expression in human synovial fibroblasts by inhibiting microRNA 519b synthesis. PLoS One. 2017; 12:e0176052. 10.1371/journal.pone.0176052 28423042 PMC5397058

[7] Dehghan M, Asgharian S, Khalesi E, Ahmadi A, Lorigooini Z. Comparative study of the effect of Thymus daenensis gel 5% and diclofenac in patients with knee osteoarthritis. Biomedicine (Taipei). 2019; 9:9. 10.1051/bmdcn/2019090209 31124455 PMC6533939

[8] Sellam J, Berenbaum F. The role of synovitis in pathophysiology and clinical symptoms of osteoarthritis. Nat Rev Rheumatol. 2010; 6:625–35. 10.1038/nrrheum.2010.159 20924410

[9] Wu MH, Tsai CH, Huang YL, Fong YC, Tang CH. Visfatin Promotes IL-6 and TNF-α Production in Human Synovial Fibroblasts by Repressing miR-199a-5p through ERK, p38 and JNK Signaling Pathways. Int J Mol Sci. 2018; 19:E190. 10.3390/ijms19010190 29316707 PMC5796139

[10] van der Kraan PM. The changing role of TGFβ in healthy, ageing and osteoarthritic joints. Nat Rev Rheumatol. 2017; 13:155–63. 10.1038/nrrheum.2016.219 28148919

[11] Kuo SJ, Hsu HC, Wang CJ, Siu KK, Hsu YH, Ko JY, Tang CH. Effects of computer-assisted navigation versus conventional total knee arthroplasty on the levels of inflammation markers: A prospective study. PLoS One. 2018; 13:e0197097. 10.1371/journal.pone.0197097 29758073 PMC5951551

[12] Nakae J, Oki M, Cao Y. The FoxO transcription factors and metabolic regulation. FEBS Lett. 2008; 582:54–67. 10.1016/j.febslet.2007.11.025 18022395

[13] Kops GJ, Dansen TB, Polderman PE, Saarloos I, Wirtz KW, Coffer PJ, Huang TT, Bos JL, Medema RH, Burgering BM. Forkhead transcription factor FOXO3a protects quiescent cells from oxidative stress. Nature. 2002; 419:316–21. 10.1038/nature01036 12239572

[14] Tran H, Brunet A, Grenier JM, Datta SR, Fornace AJ Jr, DiStefano PS, Chiang LW, Greenberg ME. DNA repair pathway stimulated by the forkhead transcription factor FOXO3a through the Gadd45 protein. Science. 2002; 296:530–34. 10.1126/science.1068712 11964479

[15] Löw P. The role of ubiquitin-proteasome system in ageing. Gen Comp Endocrinol. 2011; 172:39–43. 10.1016/j.ygcen.2011.02.005 21324320

[16] Zhao J, Brault JJ, Schild A, Goldberg AL. Coordinate activation of autophagy and the proteasome pathway by FoxO transcription factor. Autophagy. 2008; 4:378–80. 10.4161/auto.5633 18227643

[17] Zhao J, Brault JJ, Schild A, Cao P, Sandri M, Schiaffino S, Lecker SH, Goldberg AL. FoxO3 coordinately activates protein degradation by the autophagic/lysosomal and proteasomal pathways in atrophying muscle cells. Cell Metab. 2007; 6:472–83. 10.1016/j.cmet.2007.11.004 18054316

[18] Manolopoulos KN, Klotz LO, Korsten P, Bornstein SR, Barthel A. Linking Alzheimer’s disease to insulin resistance: the FoxO response to oxidative stress. Mol Psychiatry. 2010; 15:1046–52. 10.1038/mp.2010.17 20966918

[19] Akasaki Y, Hasegawa A, Saito M, Asahara H, Iwamoto Y, Lotz MK. Dysregulated FOXO transcription factors in articular cartilage in aging and osteoarthritis. Osteoarthritis Cartilage. 2014; 22:162–70. 10.1016/j.joca.2013.11.004 24269635 PMC3932989

[20] Lee RC, Feinbaum RL, Ambros V. The C. elegans heterochronic gene lin-4 encodes small RNAs with antisense complementarity to lin-14. Cell. 1993; 75:843–54. 10.1016/0092-8674(93)90529-Y 8252621

[21] Liu SC, Chuang SM, Hsu CJ, Tsai CH, Wang SW, Tang CH. CTGF increases vascular endothelial growth factor-dependent angiogenesis in human synovial fibroblasts by increasing miR-210 expression. Cell Death Dis. 2014; 5:e1485. 10.1038/cddis.2014.453 25341039 PMC4649533

[22] Asahara H. Current Status and Strategy of microRNA Research for Cartilage Development and Osteoarthritis Pathogenesis. J Bone Metab. 2016; 23:121–27. 10.11005/jbm.2016.23.3.121 27622175 PMC5018604

[23] Liu SC, Chiu CP, Tsai CH, Hung CY, Li TM, Wu YC, Tang CH. Soya-cerebroside, an extract of Cordyceps militaris, suppresses monocyte migration and prevents cartilage degradation in inflammatory animal models. Sci Rep. 2017; 7:43205. 10.1038/srep43205 28225075 PMC5320555

[24] Lin H, Li N, He H, Ying Y, Sunkara S, Luo L, Lv N, Huang D, Luo Z. AMPK Inhibits the Stimulatory Effects of TGF-β on Smad2/3 Activity, Cell Migration, and Epithelial-to-Mesenchymal Transition. Mol Pharmacol. 2015; 88:1062–71. 10.1124/mol.115.099549 26424816 PMC4658597

[25] Xu L, Zhai L, Ge Q, Liu Z, Tao R. Vacuolar Protein Sorting 4B (VPS4B) Regulates Apoptosis of Chondrocytes via p38 Mitogen-Activated Protein Kinases (MAPK) in Osteoarthritis. Inflammation. 2017; 40:1924–32. 10.1007/s10753-017-0633-2 28744712

[26] Sun HY, Hu KZ, Yin ZS. Inhibition of the p38-MAPK signaling pathway suppresses the apoptosis and expression of proinflammatory cytokines in human osteoarthritis chondrocytes. Cytokine. 2017; 90:135–43. 10.1016/j.cyto.2016.11.002 27907835

[27] Li J, Miller EJ, Ninomiya-Tsuji J, Russell RR 3rd, Young LH. AMP-activated protein kinase activates p38 mitogen-activated protein kinase by increasing recruitment of p38 MAPK to TAB1 in the ischemic heart. Circ Res. 2005; 97:872–79. 10.1161/01.RES.0000187458.77026.10 16179588

[28] Lanna A, Henson SM, Escors D, Akbar AN. The kinase p38 activated by the metabolic regulator AMPK and scaffold TAB1 drives the senescence of human T cells. Nat Immunol. 2014; 15:965–72. 10.1038/ni.2981 25151490 PMC4190666

[29] Nakasa T, Nagata Y, Yamasaki K, Ochi M. A mini-review: microRNA in arthritis. Physiol Genomics. 2011; 43:566–70. 10.1152/physiolgenomics.00142.2010 21325061

[30] Shen J, Li S, Chen D. TGF-β signaling and the development of osteoarthritis. Bone Res. 2014; 2:14002. 10.1038/boneres.2014.2 25541594 PMC4274935

[31] He L, Hannon GJ. MicroRNAs: small RNAs with a big role in gene regulation. Nat Rev Genet. 2004; 5:522–31. 10.1038/nrg1379 15211354

[32] Yang WH, Tsai CH, Fong YC, Huang YL, Wang SJ, Chang YS, Tang CH. Leptin induces oncostatin M production in osteoblasts by downregulating miR-93 through the Akt signaling pathway. Int J Mol Sci. 2014; 15:15778–90. 10.3390/ijms150915778 25198901 PMC4200751

[33] June RK, Liu-Bryan R, Long F, Griffin TM. Emerging role of metabolic signaling in synovial joint remodeling and osteoarthritis. J Orthop Res. 2016; 34:2048–58. 10.1002/jor.23420 27605370 PMC5365077

[34] Salminen A, Kaarniranta K. AMP-activated protein kinase (AMPK) controls the aging process via an integrated signaling network. Ageing Res Rev. 2012; 11:230–41. 10.1016/j.arr.2011.12.005 22186033

[35] Tang CH, Chiu YC, Tan TW, Yang RS, Fu WM. Adiponectin enhances IL-6 production in human synovial fibroblast via an AdipoR1 receptor, AMPK, p38, and NF-kappa B pathway. J Immunol. 2007; 179:5483–92. 10.4049/jimmunol.179.8.5483 17911635

[36] Ambros V. The functions of animal microRNAs. Nature. 2004; 431:350–55. 10.1038/nature02871 15372042

[37] Liu SC, Hsu CJ, Chen HT, Tsou HK, Chuang SM, Tang CH. CTGF increases IL-6 expression in human synovial fibroblasts through integrin-dependent signaling pathway. PLoS One. 2012; 7:e51097. 10.1371/journal.pone.0051097 23227240 PMC3515445

[38] Liu SC, Lee HP, Hung CY, Tsai CH, Li TM, Tang CH. Berberine attenuates CCN2-induced IL-1β expression and prevents cartilage degradation in a rat model of osteoarthritis. Toxicol Appl Pharmacol. 2015; 289:20–29. 10.1016/j.taap.2015.08.020 26344001

[39] Tang CH, Hsu CJ, Fong YC. The CCL5/CCR5 axis promotes interleukin-6 production in human synovial fibroblasts. Arthritis Rheum. 2010; 62:3615–24. 10.1002/art.27755 20862675

[40] Su CM, Hsu CJ, Tsai CH, Huang CY, Wang SW, Tang CH. Resistin Promotes Angiogenesis in Endothelial Progenitor Cells Through Inhibition of MicroRNA206: Potential Implications for Rheumatoid Arthritis. Stem Cells. 2015; 33:2243–55. 10.1002/stem.2024 25828083

[41] Wang SW, Liu SC, Sun HL, Huang TY, Chan CH, Yang CY, Yeh HI, Huang YL, Chou WY, Lin YM, Tang CH. CCL5/CCR5 axis induces vascular endothelial growth factor-mediated tumor angiogenesis in human osteosarcoma microenvironment. Carcinogenesis. 2015; 36:104–14. 10.1093/carcin/bgu218 25330803

[42] Tsai HC, Tzeng HE, Huang CY, Huang YL, Tsai CH, Wang SW, Wang PC, Chang AC, Fong YC, Tang CH. WISP-1 positively regulates angiogenesis by controlling VEGF-A expression in human osteosarcoma. Cell Death Dis. 2017; 8:e2750. 10.1038/cddis.2016.421 28406476 PMC5477571

[43] Lee HP, Chen PC, Wang SW, Fong YC, Tsai CH, Tsai FJ, Chung JG, Huang CY, Yang JS, Hsu YM, Li TM, Tang CH. Plumbagin suppresses endothelial progenitor cell-related angiogenesis in vitro and in vivo. J Funct Foods. 2019; 52:537–44. 10.1016/j.jff.2018.11.040

