# Supplementary material for: TGF-β1 enhances FOXO3 expression in human synovial fibroblasts by inhibiting miR-92a through AMPK and p38 pathways
Source: Aging (Albany NY). 2019 Jun 21;11(12):4075–89. doi: 10.18632/aging.102038 (PMC6628998; doi:10.18632/aging.102038)
Supplement: Supplementary Figures [file aging-11-102038-s001.pdf]

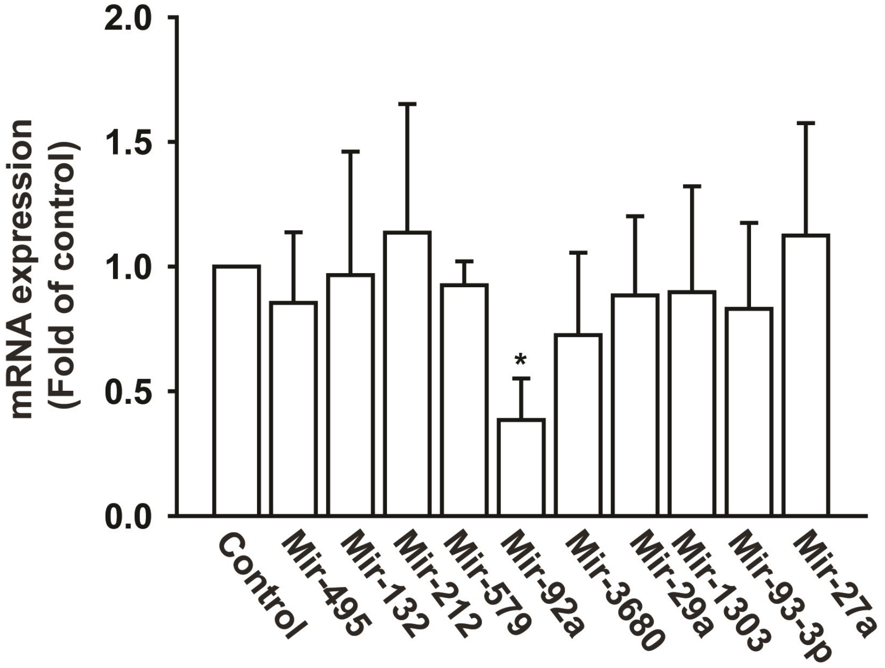

Figure S1. The TGF-β1 inhibits miR-92a expression. OASFs were incubated with TGF-β1 (10 ng/ml) for 24 h. The miRNA expression was examined by qPCR. Results are expressed as the mean ± SEM. *$p < 0.05$ as compared with the control group.

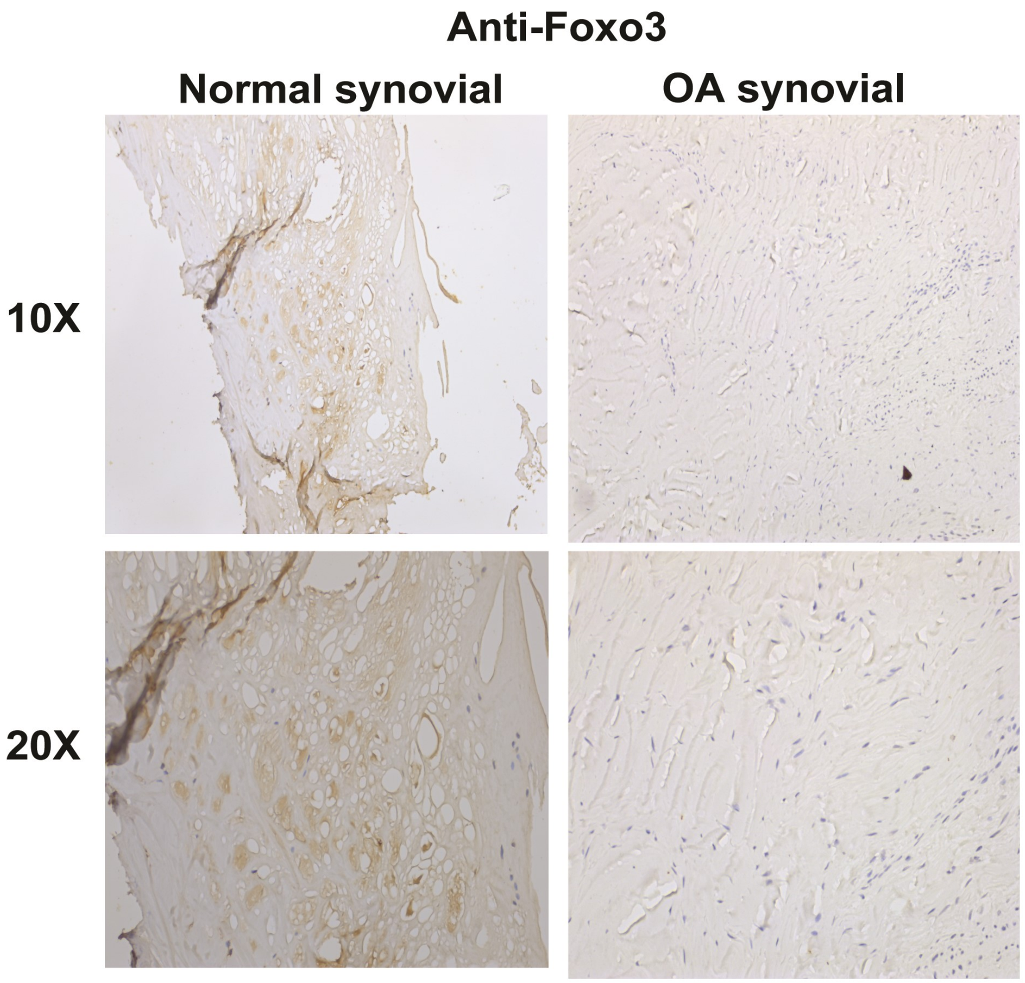

Figure S2. The FOXO3 expression in normal and OA patients. The normal and OA specimens were immunostained (IHC) with anti-FOXO3 antibody.

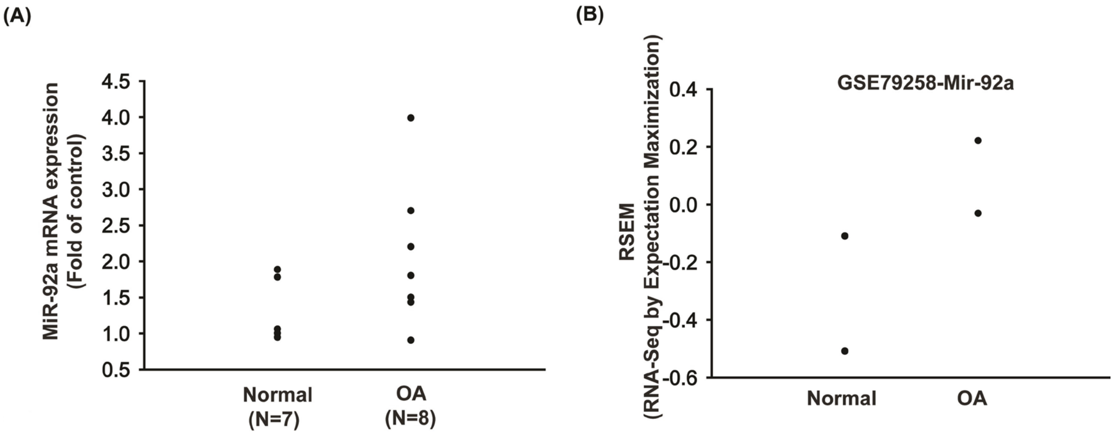

Figure S3. The miR-92a expression in normal and OA patients. (**A**) The miR-92a expression in normal and OA specimens were examined by qPCR. (**B**) Expression levels of miR-92a in paired normal and OA tissues retrieved from the GEO dataset records.
